# Initiated Chemical Vapor Deposition (iCVD) Functionalized Polylactic Acid–Marine Algae Composite Patch for Bone Tissue Engineering

**DOI:** 10.3390/polym13020186

**Published:** 2021-01-07

**Authors:** Wiebke Reichstein, Levke Sommer, Salih Veziroglu, Selin Sayin, Stefan Schröder, Yogendra Kumar Mishra, Eyüp İlker Saygili, Fatih Karayürek, Yahya Açil, Jörg Wiltfang, Aydin Gülses, Franz Faupel, Oral Cenk Aktas

**Affiliations:** 1 Chair for Multicomponent Materials, Institute of Materials Science, Faculty of Engineering, Kiel University, Kaiserstr. 2, 24143 Kiel, Germany; wire@tf.uni-kiel.de (W.R.); sve@tf.uni-kiel.de (S.V.); ssch@tf.uni-kiel.de (S.S.); ff@tf.uni-kiel.de (F.F.); 2 Department of Oral and Maxillofacial Surgery, Campus Kiel, University Hospital of Schleswig-Holstein, Arnold-Heller-Straße 3, 24105 Kiel, Germany; levke-sommer@live.de (L.S.); Yahya.Acil@uksh.de (Y.A.); Joerg.Wiltfang@uksh.de (J.W.); 3 Marine Science and Technology Faculty, Iskenderun Technical University, 31200 Iskenderun/Hatay, Turkey; selin.sayin@iste.edu.tr; 4 Mads Clausen Institute, NanoSYD, University of Southern Denmark, Alsion 2, 6400 Sønderborg, Denmark; mishra@mci.sdu.dk; 5 Department of Medical Biochemistry, SANKO University, Şehitkamil, 27090 Gaziantep, Turkey; ISaygili@sanko.edu.tr; 6 Department of Periodontology, Cankiri Karatekin University, 18100 Cankiri, Turkey; fatihkarayurek@karatekin.edu.tr

**Keywords:** PLA, hydrogel, iCVD, osteoblast, biocompatibility, proliferation, tissue engineering

## Abstract

The current study aimed to describe the fabrication of a composite patch by incorporating marine algae powders (MAPs) into poly-lactic acid (PLA) for bone tissue engineering. The prepared composite patch was functionalized with the co-polymer, poly (2-hydroxyethyl methacrylate-co-ethylene glycol dimethacrylate) (p(HEMA-co-EGDMA)) via initiated chemical vapor deposition (iCVD) to improve its wettability and overall biocompatibility. The iCVD functionalized MAP–PLA composite patch showed superior cell interaction of human osteoblasts. Following the surface functionalization by p(HEMA-co-EGDMA) via the iCVD technique, a highly hydrophilic patch was achieved without tailoring any morphological and structural properties. Moreover, the iCVD modified composite patch exhibited ideal cell adhesion for human osteoblasts, thus making the proposed patch suitable for potential biomedical applications including bone tissue engineering, especially in the fields of dentistry and orthopedy.

## 1. Introduction

In dentistry and orthopedy, bone tissue engineering has come to the fore in recent years with new approaches to treat bone insufficiency or defects arising from a tumor, trauma, or periodontal diseases [1]. In general, bone tissue engineering requires artificial materials with physicochemical, structural, and biological properties that promote cellular attachment, proliferation, and differentiation [2,3]. Various types of materials including metals, bioactive ceramics and glasses, natural and synthetic polymers, and their composites have been assessed and utilized as scaffolds for successful bone regeneration [4]. Among them, polymers and their composites are considered as the most promising candidates due to their proven biocompatibility over most metals and ceramics [5].

Besides various types of polymers, aliphatic polyesters are the preferred substances for bone tissue engineering applications essentially owing to their bioresorbable nature [6]. Polylactic acid (PLA), polyglycolic acid (PGA), poly-ε-caprolactone (PCL), and their copolymers are especially frequently used in the fabrication of scaffolds, membranes, and patches in bone tissue engineering [7]. PLA-based biomaterials are considered to be the gold standard for various regenerative engineering applications because of their superior biodegradability, and compatibility with biomolecules and cells [8,9]. In addition, PLA-based biomaterials have the ability to be fabricated into a variety of structures (planar membranes, 3D scaffolds, fibers, etc.) with the appropriate mechanical properties, topography, geometry, and architecture [10].

In recent years some studies have proclaimed that composites prepared by blending biodegradable synthetic polymers with biopolymers or their biological sources, for instance algal biomass, exhibit superior and add-on properties [11,12,13,14]. Marine algae are primarily abundant in the junctures between ocean and land and can be collected in the coastal areas of various countries [15]. Marine algae also have a high nutrient content and contain several biologically active components such as pigments/carotenoids, vitamins, and antioxidants [16]. PLA-based synthetic polymers blended with marina algae powders (MAPs) have been shown to exhibit significantly improved cytocompatibility [17]. Wu et al. reported that the incorporation of MAPs in polyesters enhances biodegradability, which is also an essential issue in bone tissue engineering [18]. In addition, blending MAPs with synthetic polyesters significantly improves the overall mechanical properties; for example, Bulota et al. showed that PLA–MAP composite has at least a 40% higher Young’s modulus as compared with the neat PLA [19].

Recently, a new type of a composite patch prepared by blending PLA with MAPs as an alternative to porcine-derived collagen membranes has been reported [20]. While the vegan nature of the PLA–MAP composite membrane provides an extreme advantage over commercial animal-derived ones (there is a risk of transmission of infection from the cells or the tissues of the graft during the microbiological screening of source animals), it exhibits a very poor level of wettability with a water contact angle (WCA) exceeding 85°. For instance, Liu et al. showed that the WCA of pristine PLA may exceed 125°, which may negatively influence cellular attachment and proliferation [21]. According to the literature, the wetting angle properties of biomaterials could be improved by initiated chemical vapor deposition (iCVD) coatings. A recent article has shown that hydrophilic gelatin nanofibers became hydrophobic after iCVD coating and might be appropriate options in biomedical applications, for instance, tissue engineering scaffolds and wound dressings [22].

In this current work, the fabrication of a composite patch by incorporating marine algae into PLA for bone tissue engineering has been described. Following the surface functionalization by poly (2- hydroxyethyl methacrylate-co-ethylene glycol dimethacrylate) (p(HEMA-co-EGDMA)) via the iCVD technique, a highly hydrophilic patch was achieved without tailoring any morphological and structural properties. Moreover, the iCVD modified composite patch exhibited ideal cell adhesion for human osteoblasts (HOBs) thus making the proposed patch suitable for potential biomedical applications including bone tissue engineering in dentistry and orthopedy.

## 2. Materials and Methods

### 2.1. Preparation of PLA-Based Composite Patch and Coating by iCVD

**Figure 1:**
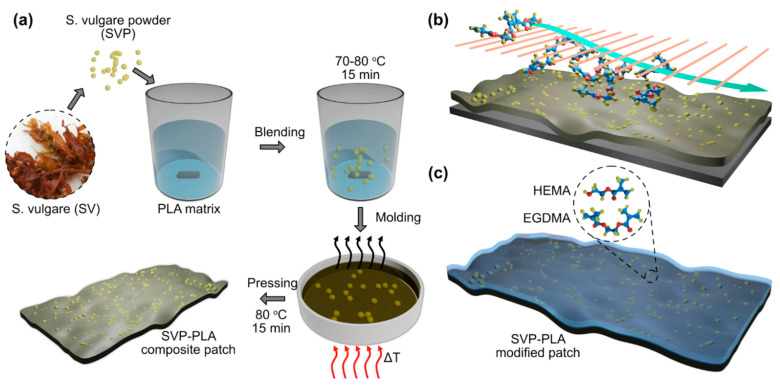
A schematic depiction of: (**a**) the preparation of SVP–PLA patch from marine algae; (**b**) the surface modification of the SVP–PLA patch with a p(HEMA-co-EGDMA) layer via iCVD, and (**c**) a p(HEMA-co-EGDMA) coated SVP–PLA modified patch.

The mentioned composite structures were prepared by a two-step processing (schematically presented in Figure 1: (i) synthesis of PLA-based composite by blending low molecular PLA with MAPs to enhance the cytocompatibility and cell viability and (ii) functionalization of the composite patch with the co-polymer, poly(2-hydroxyethyl methacrylate-co-ethylene glycol dimethacrylate) (p(HEMA-co-EGDMA)) via initiated chemical vapor deposition (iCVD) to improve the wettability and overall biocompatibility.

Following the cleaning and the purification of *S. vulgare* (a type of brown algae) seaweed specimens (which were collected from the Mediterranean Sea in May 2020), were ground and passed through 300- and 400-mesh sieves, air-dried for 1 day at 50–60 °C, and vacuum-dried for at least 12 h at 105–110 °C until the moisture content of the resulting fine, the brown powder was 3 ± 0.5%. The composites were prepared by mixing functionalized MAPs with PLA (at a mass ratio of 15:85) at 70–80 °C for 15 min using a mechanical mixer operating at a speed of 50–60 rpm. After mixing, the PLA–MAP blend was poured into a Teflon mold and hot-pressed while the plate temperature was gradually increased from 50 °C to 85 °C for 15 min. Controlled evaporation of the solvent and applied pressure led to the formation of a porous patch as seen in Figure 1a.

A scroll pump (nXDS 10i Edwards, Burgess Hill, UK) was used to evacuate the vacuum chamber. The monomers, HEMA (97%, abcr GmbH, Karlsruhe, Germany), and EGDMA (98%, abcr GmbH, Karlsruhe, Germany) were heated in glass jars to 75 °C and their vapors were fed into a custom-made hot filament CVD reactor. Their respective flows were regulated with the help of flow metering valves (Swagelok). For the lower crosslinked coatings, HEMA flow was set to 0.3 sccm and EGDMA flow to 0.1 sccm and vice versa for the high-density layers. Here the density refers to the cross-linking degree of the deposited co-polymer. The initiator Tert-butyl-peroxide (TBPO, 95%, Fluorochem Ltd., Hadfield, UK) is held at room temperature. The flow was set to 0.6 sccm and regulated with a flow metering valve (Vögtlin Instruments GmbH, Muttenz, Switzerland). Furthermore, a constant nitrogen patch flow of 0.3 sccm was introduced into the chamber via a mass flow controller (MC series, Alicat Scientific Inc., Tucson, AZ, USA). During the deposition, the substrate temperature was kept at 30 °C. A butterfly valve (VAT 615), together with a capacitive monometer (MKS Baratron), held the process pressure at 40 Pa. The filament power was P = 40 W. Modification of *S. vulgare* powder- polylactic acid (SVP–PLA) composite patch with p(HEMA-co-EGDMA) is schematically depicted in Figure 1c.

### 2.2. Materials Characterization

Fourier-transform infrared (FTIR) spectra of dry samples have been acquired with a Bruker Vertex 80v spectrometer (Bruker, Billerica, MA, USA) operating in the range of 1000 cm^−1^ and 4000 cm^−1^. The baseline correction was done using built-in software. Fluorescein diacetate (FDA, Sigma- Aldrich, St. Louis, MO, USA) and propidium iodide (PI, Sigma-Aldrich, St. Louis, MO, USA) were used. A semi-automated contact angle meter (OCA 30, Dataphysics, Filderstadt, Germany) was performed for wetting contact angle (WCA) measurements. After this, 10 µL of water droplets were used and advancing/receding CAs were recorded and measured by the addition and subtraction of water to/from droplets sitting on the sample surface. The cells were assessed with a fluorescence microscope (Axioplan2) and documented with a digital camera (AxioCam MRc5 from ZEISS, Oberkochen, Germany). The dyes could be excited at 488 nm (blue light, argon laser). The green fluorescence (FDA) was detected at 530 nm.

### 2.3. Cytocompatibility Analysis

The study was embarked upon after receiving approval from The Ethics Committee of the Medical Faculty of Christian Albrechts University, Kiel, Germany. (D640/20) and was conducted according to the guidelines of the Helsinki Declaration of Human Rights. Osteoblast cultivation was conducted according to the technique described by Naujokat et al. [23] Human osteoblasts were obtained from patients who had undergone bone graft harvesting procedures of cancellous bone from the crista iliaca anterior at the Oral and Maxillofacial Surgery Department at the Christian Albrechts University Hospital Schleswig-Holstein, Kiel Campus, Kiel, Germany. The osseous samples were transferred into the 89% Dulbecco’s Modified Eagle’s Minimum Essential Medium (DMEM) (PAA Laboratories GmbH, Pasching, Austria), 10% fetal calf serum (FCS) (Biochrom, Berlin, Germany, 1% Penicillin/Streptomycin, Biochrom, Berlin, Germany). Further processing took place under laminar flow (Heraeus Instruments, Osterode, Germany). Then, they were minced into ∼1–2 mm pieces and placed into cell culture flasks (Thermo-Fisher Scientific, Waltham, MA, USA) containing 10 mL cell culture medium. The osteoblasts were passaged after reaching 80% confluence. Following aspiration of the medium and concomitant rinsing with 10 mL of phosphate-buffered solution (PBS) (Sigma-Aldrich, St. Louis, MO, USA), 5 mL of PBS containing 0.05% trypsin was added to each culture flask to detach and remove the osteoblasts from the surface, followed by the dilution of the cell suspension via DMEM enriched with 10% FCS to inhibit the action of trypsin. After that, the cell suspension was centrifuged at 3200 rounds per minute (rpm) for 180 s. The supernatant was filtered, suctioned off and the remaining cell pellet was resuspended in 5 mL of medium and the cells were counted in a Neubauer counting chamber (Brand, Wertheim, Germany), after which 10^5^ osteoblasts were transferred into a 75 cm^3^ culture flask containing 10 mL of medium. The incubation process was conducted with eluates, as well as indirect contact with scaffolds.

### 2.4. MTT Assay

The proliferation of the osteoblasts was evaluated via an MTT Cell Proliferation Kit (11465007001, Roche Diagnostics, Mannheim, Germany). We incubated 96-well microtiter plates with 5 × 10^3^ cells/well for 24 h. After that, a sample of 100 μL eluate was obtained. Following an incubation for 24 h, cell proliferation was quantified. The optical density of the samples was examined photometrically at a wavelength of 450 nm.

### 2.5. BrdU Assay

The osteoblast proliferation rate was determined by using the BrdU (Bromodeoxyuridine) Cell Proliferation enzyme-linked immunoadsorption tool (Roche Diagnostics, Mannheim, Germany). Similar to the MTT assay, 96-well microtiter plates with 5 × 10^3^ cells/ well were incubated for 24 h and a sample of 150 μL eluate was obtained. After 48 h, the eluate was changed to a standard nutrient medium and the osteoblasts were incubated for another 72 h. After that, 10 μL of BrdU solution was added to each well and the osteoblasts were incubated for an additional 24 h so that the osteoblasts could incorporate bromodeoxyuridine into their DNA. The optical density of each sample was examined in a microplate reader (Spectra Max plus 384, Molecular Devices, Sunnyvale, CA, USA) at a wavelength of 450 nm.

## 3. Results

**Figure 2:**
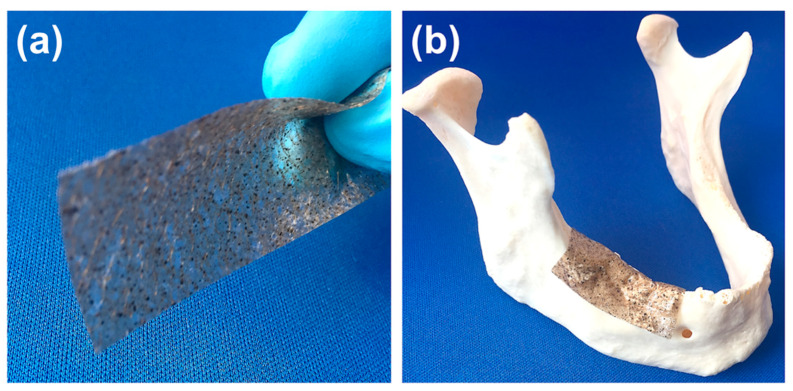
(**a**) An SVP–PLA composite patch and (**b**) the use of SVA–PLA composite patch in the reconstruction of maxillofacial osseous defects.

This work describes the fabrication of PLA-based porous composite structures with superior wetting and bioactive properties, which opens up the possibility of using them as a membrane or patch, especially in the reconstruction of maxillofacial osseous defects as shown in Figure 2.

### 3.1. FTIR Analysis and Wetting Properties

**Figure 3:**
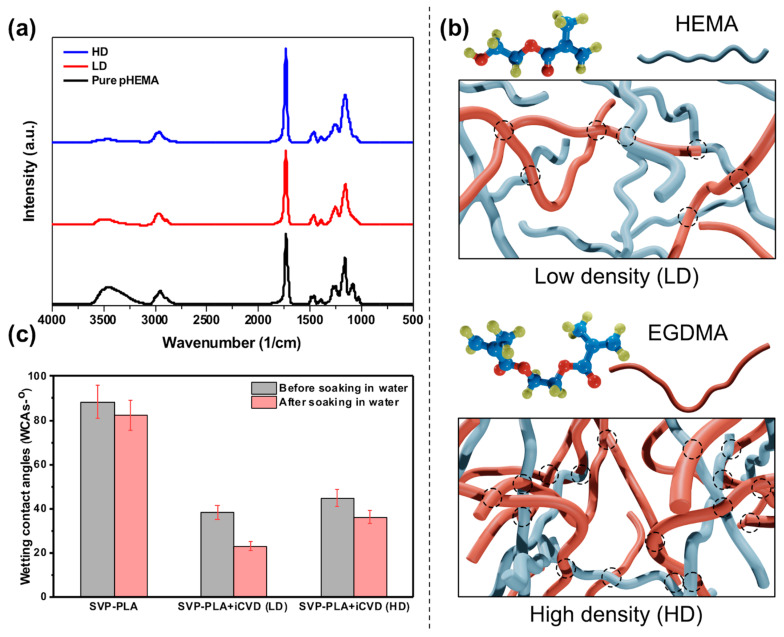
(**a**) FTIR spectra of prepared coatings; (**b**) a schematic description for low- and high-density p(HEMA-EGDMA) layers (dashed circles indicate cross-linking); and (**c**) The water contact angles (WCAs) of prepared samples before and after soaking in water.

p(HEMA-co-EGDMA) layer was coated on both sides of SVP–PLA patches to obtain a homogenous film thickness around 175–200 nm. Afterward, Fourier-transform infrared (FTIR) spectroscopy analysis was performed to determine the exact film composition of the p(HEMA-co-EGDMA) layer. Here, a FTIR spectrometer (Vertex 80v, Bruker, Brillerica, MA, USA) in transmission mode from 500 to 4000 cm⁻^1^ at 4 cm⁻^1^ step width was used. The crosslinking degree can be calculated from the respective spectra of high density (HD) and low density (LD) films by taking the ratios of the respective characteristic peak areas of HEMA and EGDMA (Figure 3a). The OH– band (3150–3600 cm^−1^) is characteristic for HEMA as well as the ester peak at 1730 cm^−1^. For EGDMA the ester peak is characteristic as well, however, the intensity is higher due to the presence of two ester groups in the monomer structure (see the structure of monomers in Figure 3b). The intensity of the ester peak is increasing whereas the peak of the hydroxyl group is decreasing with increasing EGDMA content. By taking this into account one gets the following formula used to calculate the composition of the co-polymer [24]:

(1) $$\frac{EGDMA}{HEMA}=\;\frac{\frac{1}{2}\left(A_{C=O}-rA_{OH}\right)}{rA_{OH}}$$

where *A* represents the respective characteristic areas under the peaks and *r* corresponds to the ratio of the peaks of the homopolymer HEMA. The calculations reveal crosslinking degrees of 57.1% LD and 80.4% for HD samples, respectively (the structure of the LD and HD p(HEMA-co-EGDMA) layers are schematically depicted in Figure 3b for comparison).

**Figure 4:**
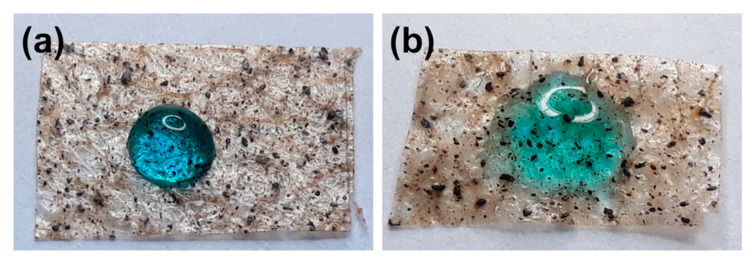
The behavior of water drops on: (**a**) the SVP–PLA patch and (**b**) the p(HEMA-co-EGDMA) coated SVP–PLA modified patch.

As shown in Figure 3c, the WCA was 88.6 ± 7.3° for the as-prepared SVP–PLA composite, and iCVD modified patches exhibited low WCAs of 38.5 ± 3.2° and 45 ± 3.8° for LD and HD p(HEMA-co-EGDMA), respectively. When the samples were soaked in water, it was observed that the WCAs decreased significantly. While the as-prepared SVA–PLA patch exhibited a WCA of 82.5 ± 7.0°, the WCAs of the control substrate and the as-prepared SVP–PLA patch-modified with LD and HD were 23.3 ± 1.9° and 36.4 ± 3.1°, respectively. It has been reported that when HEMA is exposed to air, the hydrophobic methyl groups become directed toward air due to interface caused by chain rotation (which means the reorientation of its hydrophilic groups toward the water). As a result of the ∼100% retention of the hydroxyl (–OH) functional groups from the HEMA monomer, iCVD p(HEMA-co-EGDMA) seems to add a hydrophilic behavior to the SVP–PLA patch. Actually, without any detailed analysis of WCAs, one can clearly see that p(HEMA-co-EGDMA) functionalized SVP–PLA tends to be wetted easily by the water droplets (Figure 4). This may provide a suitable microenvironment for the attachment and proliferation of cells because they have become more hydrophilic.

### 3.2. MTT Assay

**Figure 5:**
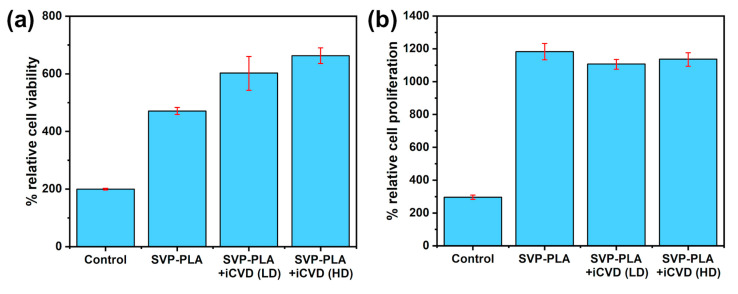
(**a**) MTT and (**b**) BrdU assays at 48 h.

The adhesion and proliferation of Human Osteoblasts (HOBs) was investigated to evaluate whether the p(HEMA-co-EGDMA) modified SVP–PLA patch satisfied the basic requirements for bone tissue engineering. The first cytotoxicity and viability of HOBs were investigated by an MTT assay. As shown in Figure 5a the metabolic activity (OD value) of HOBs on SVP–PLA and SVP–PLA + iCVD (LD; p(HEMA-co-EGDMA) and HD; p(HEMA-co-EGDMA)) was significantly higher compared to the control group (*p* < 0.005). Despite higher cell viability of both iCVD coated SVP–PLA patches, the difference between SVP–PLA and SVP–PLA + iCVD (LD; p(HEMA-co-EGDMA) and HD; p(HEMA-co-EGDMA)) was statistically insignificant. (*p* > 0.005) The enhancement of the cell viability could be attributed to the improved hydrophilicity of iCVD, which might have enhanced the protein adsorption capacity of a surface and promoted cellular behaviors, including the initial attachment, proliferation, and differentiation. Here one should keep in mind that the improvement in cell viability can be also correlated with the extremely low cytotoxicity of HEMA and its co-polymers.

### 3.3. BrdU Assay

The proliferative behavior of the prepared surfaces was tested using the standard BrdU assay. Basically, the BrdU assay is based on the ability of proliferating cells to incorporate the BrdU reagent into their DNA as they add thymidine during DNA replication and synthesis [25]. The results suggested significantly higher proliferation rates of HOBs on all SVP–PLA patches, compared to the control group. The difference between SVP–PLA, SVP–PLA + iCVD (HD) and SVP–PLA + iCVD (LD) was statistically insignificant (*p* > 0.0005) (Figure 5b).

### 3.4. Fluorescein-Diacetate Analysis

Fluorescence microscopic observation was conducted after 48 h of culture. Fluorescein diacetate (FDA, Sigma- Aldrich, St. Louis, MO, USA) and propidium iodide (PI, Sigma-Aldrich, St. Louis, MO, USA) were used. The cells were assessed with the fluorescence microscope (Axioplan2) and documented with a digital camera (AxioCam MRc5 from ZEISS, Oberkochen, Germany). The dyes could be excited at 488 nm (blue light, argon laser). The green fluorescence (FDA) was detected at 530 nm.

**Figure 6:**
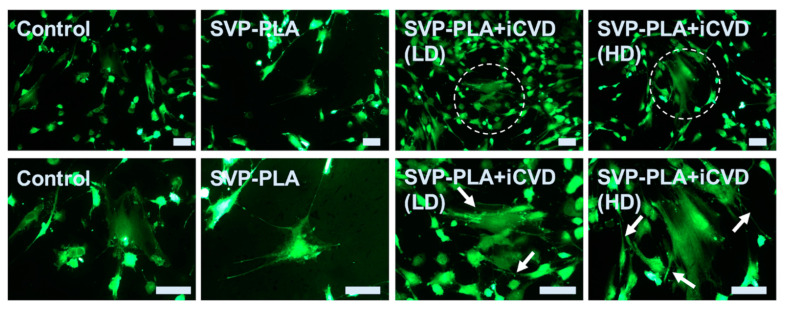
Fluorescence imaging of Human Osteoblasts (HOBs) on the control substrate and prepared patches at 48 h cell culturing. (All scale bars correspond to 20 µm).

Figure 6 shows adherent HOBs cultured on the control substrate and prepared patches. The results indicated a well-attached and elongated morphology, which is a major sign of viable cells. However, there was a significant difference in the numbers of cells, especially for those cultured on p(HEMA-co-EGDMA) modified and as-prepared patches. FDA analysis revealed higher cell numbers of both HD and LD iCVD coatings compared to the SVP–PLA and control groups, which were also correspondent with the MTT results. However, regardless of the cell numbers, on both p(HEMA-co-EGDMA) modified patches (LD and HD) and HOBs revealed more cell-to-cell interaction (indicated by the dashed circle) compared to the control substrate and as-prepared SVP–PLA patches. Additionally, cell density has clearly increased on the p(HEMA-co-EGDMA) modified patches. At higher magnifications, it could be observed that HOBs started to form a dense network of filopodia (indicated by arrows). Cell-to-cell contacts were established and HOBs exited an extremely spread morphology. On the contrary, one can see that HOBs exhibited a totally different morphology on the control substrate. Despite the similar proliferation rates of HOBs on SVP–PLA and p(HEMA-co-EGDMA) modified patches (LD and HD) observed from BrdU test, the morphology of the cells on an as-prepared SVP–PLA patch was prone to indicate a non-spreading characteristic with lesser filopodia-like extensions.

## 4. Discussion

The hydrophilicity of the materials plays an important role in their interactions with the cells [26]. The WCAs of prepared SVP–PLA patches were measured before and after the surface functionalization by iCVD to evaluate whether the hydrophilicity was improved by the deposition of the p(HEMA-co-EGDMA) co-polymer. Additionally, the WCAs of prepared samples were investigated before and after soaking them in water. It is known that by increasing the incorporation of EGDMA, the swelling of the p(HEMA-co-EGDMA) films in water is tunable from ∼25% for pure HEMA down to ∼0% for pure EGDMA [27]. In general, when a polymer surface is exposed to a dry atmosphere, the change in the surface configuration promotes a more hydrophobic state [28]. When the surrounding medium is changed from the dry atmosphere to an aqueous medium, the hydrophilicity increases significantly.

Xeno-based collagen materials have been widely used as bioresorbable barrier membranes in guided tissue/bone regeneration. However, in addition to the controversies regarding the risk of the transmission of infection by the cells or the tissues of the graft during the microbiological screening of source animals, infections at surgical sites present a realistic challenge for guided tissue/bone regeneration [29]. Moreover, it is very well known that collagen, which exhibits rapid biodegradation accompanied by a notable decrease of mechanical stability in the human body, is one of the most popular biomedical materials. However, this fact represents the key challenge for its use in large-sized tissue regeneration, which takes a long time [30]. Therefore, numerous studies aimed to improve the biomechanical characteristics of those membranes via nano-material applications [31]. On the other hand, the aim of the current study was to improve the hydrophilic characteristics of the recently described marine-algae composite patches by iCVD modification to obtain superior osteoblastic proliferative behavior. However, there is still a need for further studies to identify their biodegradation and biomechanical stability.

It has been previously proclaimed that alginate membranes may be a useful alternative to collagen-based membranes when the Guided bone regeneration (GBR) is employed. Moreover, alginate membranes were also proposed as a self-setting barrier membrane that can be used for GBR [32]. Functionalized polymer nanolayer deposition via iCVD is a flexible and robust technique capable of the mass production of biocompatible layers, and is suggested to be a very suitable modification method in biomedical engineering [33]. In recent years, there has also been a growing interest in iCVD modified bio-patches, thus they can enable antibacterial and biocompatible surfaces [34]. The iCVD modified composite patch presented herein exhibited ideal cell adhesion for human osteoblasts, thus making the proposed patch suitable for potential biomedical applications including bone tissue engineering, especially in the fields of dentistry and orthopedy. However, further studies are needed to clarify the fibroblastic proliferation and to speculate on its suitability for GBR applications. On the other hand, its anti-bacterial characteristics could also be the subject of future studies.

## 5. Conclusions

This work demonstrates the fabrication of a composite patch by incorporating marine algae into PLA for bone tissue engineering. Following functionalization of the surface by p(HEMA-co-EGDMA) via the iCVD technique, a highly hydrophilic patch was achieved without tailoring any morphological or structural properties. Moreover, the iCVD modified composite patch exhibited ideal cell adhesion for HOBs, thus making the proposed patch suitable for potential biomedical applications including bone tissue engineering in dentistry and orthopedy. However, further studies, such as the promotion of bone mineral deposition after surface functionalization via the iCVD technique, are needed to exactly define the bone cells’ behavior.

## Data Availability

The data presented in this study are available on request from the corresponding author.
